# Mammalian Reoviruses: Propagation, Quantification, and Storage

**DOI:** 10.1002/cpz1.716

**Published:** 2023-04-11

**Authors:** Kevin M. Coombs

**Affiliations:** ^1^ University of Manitoba and Manitoba Centre for Proteomics and Systems Biology Winnipeg Manitoba Canada

**Keywords:** cesium chloride ultracentrifugation, double‐stranded RNA, mouse cell culture, non‐enveloped virus, plaque assay, RNA virus

## Abstract

Mammalian reoviruses are pathogens that cause gastrointestinal and respiratory infections. In humans, the mammalian reoviruses usually cause mild or subclinical disease, and they are ubiquitous, with most people mounting immunity at a young age. Reoviruses are prototypic representations of the *Reoviridae* family, which contains many highly pathogenic viruses. This article describes techniques for culturing mouse fibroblast L929 cell lines, the preferred cell line in which most mammalian reovirus studies take place. In addition, mammalian reovirus propagation, quantification, purification, and storage are described. © 2023 The Authors. Current Protocols published by Wiley Periodicals LLC.

**Basic Protocol 1**: Propagation of mammalian reoviruses in cell culture from virus stocks

**Alternate Protocol 1**: Large‐scale propagation (and purification) of mammalian reoviruses in cell culture from virus stocks

**Basic Protocol 2**: Quantification of mammalian reoviruses by plaque assay with neutral red staining

**Alternate Protocol 2**: Quantification of mammalian reoviruses by plaque assay with crystal violet staining

**Basic Protocol 3**: Storage of mammalian reoviruses

**Support Protocol 1**: Growth and maintenance of mouse L929 cells

**Support Protocol 2**: Plating L929 cells

## INTRODUCTION

Mammalian reoviruses are pathogens that present as gastrointestinal and respiratory infections. In humans, the mammalian reoviruses usually cause mild or subclinical disease, and they are ubiquitous, with most people mounting immunity at a young age. Reoviruses are prototypic representations of the *Reoviridae* family, which contains many highly pathogenic viruses. Studies with reoviruses can provide insight into these more dangerous members.

This article describes techniques on culturing mouse fibroblast L929 cell lines, in which most of the mammalian reovirus studies take place (Support Protocol 1), as well as propagating, quantifying, and storing reovirus. Basic Protocol 1 describes the propagation of reovirus in mouse L929 cell lines. The titration, or quantification, of the virus is described in Basic Protocol 2. Methods for virus storage are explained in Basic Protocol 3.

*CAUTION*: Mammalian reoviruses are Biosafety Level 2 (BSL‐2) pathogens. Follow all appropriate guidelines and regulations for the use and handling of pathogenic microorganisms.

## Basic Protocol 1 PROPAGATION OF MAMMALIAN REOVIRUSES IN CELL CULTURE FROM VIRUS STOCKS

Although mammalian reoviruses will grow in many different cell types, mouse L929 cells are preferred to use for in vitro cell culture and manipulation because the virus grows to high titer, and these cells are easily manipulated in suspension and monolayer forms. The propagation of reovirus stocks is performed by a series of passages. Typical virus passaging is described below.

### Materials

Mouse L929 cells (ATCC #CCL‐1)
Neutral red–stained plate containing virus plaques (see Basic Protocol 2)
Gel saline (see recipe)
25‐cm^2^ tissue culture flask with pre‐attached L929 cell monolayer (see Support Protocol 1)
Complete Joklik's Minimum Essential Medium (J‐MEM) (see recipe)
Penicillin‐streptomycin solution, 100× (see recipe)
Amphotericin B working solution, 1000× (see recipe)
75‐cm^2^ tissue culture flask(s) with pre‐attached L929 cell monolayer (see Support Protocol 1)

Cotton‐plugged Pasteur pipets, sterile
1‐ or 2‐dram glass vials, sterile

Additional reagents and equipment for obtaining neutral red–stained plates containing virus plaques (Basic Protocol 2)

*NOTE*: All solutions and materials that come into contact with cells must be sterile, with proper aseptic techniques used.

*NOTE*: All cell culture incubations utilize a 37°C incubator with 5% CO_2_ unless otherwise stated.

#### P_0_ (passage zero)

**Figure 1:**
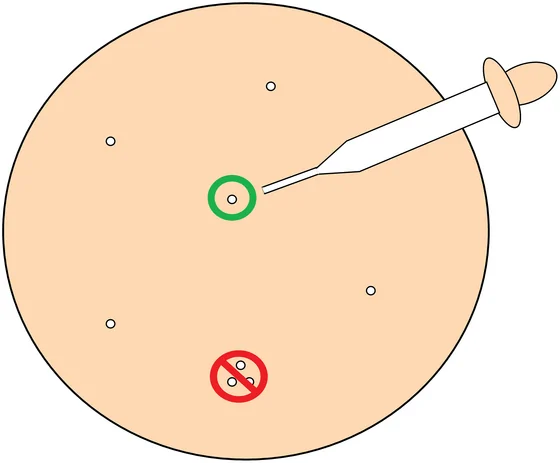
Picking virus plaques from agar plates. Well separated plaques, such as depicted in the green circle, should be picked whereas plaques close to each other (red circle) should be avoided.

1 To pick a virus plaque from a neutral red–stained plate (see Basic Protocol 2):
  - Squeeze the rubber bulb on a sterile cotton‐plugged Pasteur pipet to expel the air.
  - Insert the tip of the pipet through the agar overlay into the plaque area, until the pipet touches the cell monolayer.
  - Rotate the pipet while slowly releasing the rubber bulb to suck up the agar and cells in the selected area.
    To ensure a pure virus clone, pick only plaques that are separated from other plaques on the agar plate by ≥1 cm (Fig. 1).

2 Expel the picked plaque into 1 ml of gel saline in a sterile 1‐ or 2‐dram glass vial.
  It is generally advisable to pick three to five plaques (in case spontaneous random mutants are present) and screen each clone to ensure plaques represent parental viruses.
3 Store P_0_ virus stock at 4°C for ≥8 hr to allow virus to diffuse out of agar plug.

#### P_1_ (first passage)

4 Pour out medium from a 25‐cm^2^ tissue culture flask that contains a pre‐attached L929 cell monolayer.
5 Inoculate 0.5 ml of P_0_ virus stock into the flask.
6 Incubate the flask 1 hr at room temperature to allow virus to adsorb.
  Gently rock the flask every 10 to 12 min to disperse liquid overlay and prevent any regions of the monolayer from drying out.
7 Add 4 ml of fresh complete J‐MEM (supplemented with 40 µl 100× penicillin/streptomycin solution and 4 µl 1000× amphotericin B working solution) to the flask.
8 Incubate the flask at 37°C until ∼85% cytopathic effect (CPE).
  If using plug‐seal cap flasks, ensure cap is loose to allow gas exchange. Examine flask daily and "beat" flask (by rapping against hand) every few days to disperse infected cells.
  IMPORTANT NOTE: If using plug‐seal cap flasks, tighten cap to ensure it does not fall off during rapping step!
9 Freeze‐thaw three times by alternately placing the flask at less than –70°C and at room temperature for three complete cycles.
10 Transfer the sample into a labeled 2‐dram vial and store at 4°C.

#### P_2_ (second passage)

11 Infect a pre‐attached L929 cell monolayer in one or more 75‐cm^2^ tissue culture flask(s) with 0.5 ml of P_1_ (from step 10). Use essentially the same procedure as for P_1_ passage, except add 11 ml of complete J‐MEM per flask and store either in multiple 2‐dram vials or in larger containers (i.e., small sterile media bottles).
  The virus stocks can then be titrated to determine the amount of virus (see Basic Protocol 2). Plaques produced from the titration can serve as fresh P_0_ to replenish stocks. Stocks are stable at 4°C for long periods of time (i.e., years).

## Alternate Protocol 1 LARGE‐SCALE PROPAGATION (AND PURIFICATION) OF MAMMALIAN REOVIRUSES IN CELL CULTURE FROM VIRUS STOCKS

To prepare large amounts of purified virus, a different method of amplification (compared to Basic Protocol 1), followed by purification of the virus can be used. This is demonstrated in the following protocol.

### Materials

6.5 × 10^8^ suspension‐grown L929 cells (see Support Protocol 1)
Complete J‐MEM (see recipe)
Mammalian reovirus P_2_ stock with titer > 10^8^ PFU/ml (see Basic Protocol 1)
Penicillin‐streptomycin solution, 100× (see recipe)
Amphotericin B working solution, 1000× (see recipe)
Bleach (Hypochlorite)
HO buffer (see recipe)
10% (w/v) sodium desoxycholate (DOC)
Vertrel‐XF (Dupont Chemicals)
1.2 g/ml and 1.44 g/ml cesium chloride gradient(s) (see recipes)
Dialysis buffer (see recipe)
Glycerol (optional)

250‐ml conical centrifuge bottles
Refrigerated low‐speed (i.e., up to 2,000 rpm, or ∼850 × *g*) centrifuge with rotor for 250‐ml conical centrifuge bottles
Glass roller bottle with sterile magnetic stir bar
Pipets and pipet tips
33°C spinning water bath (spinning water bath is set up by placing a large flat‐bottom water‐proof acrylic container on top of a multi‐magnetic stirrer; the container should have sufficient room to hold a temperature‐settable immersion heater and one or more glass roller bottles)
30‐ml COREX centrifuge tubes
Ice bucket and ice
Sonicator with small probe (for ultrasonic disruption of cells)
Vortex
Parafilm
Refrigerated Super‐speed (i.e., up to 15,000 rpm, or ∼21,000 × *g*) centrifuge with rotor and adaptors for 30‐ml COREX tubes
SW‐28 "ultra‐clear"‐type ultracentrifuge tubes
Refrigerated ultracentrifuge (i.e., up to 25,000 rpm, or ≥87,000 × *g*) with swinging‐bucket rotor for SW‐28 ultracentrifuge tubes
Dialysis tubing (e.g., Spectra/por 50,000 MWCO) and clips
UV spectrophotometer
Hemocytometer

*NOTE*: These protocols are for each 1‐liter infection. They can be scaled up or down. For example, we have carried out 6‐liter infections in 12‐liter Florence flasks.

#### Perform virus propagation

1 Pour 6.5 × 10^8^ suspension‐grown L929 cells into 250‐ml centrifuge bottles.
2 Spin cells in a refrigerated centrifuge 12 min at ∼350 × *g*, 4°C.
3 Pour 250 ml of the supernatant into the glass roller bottle (use supernatant as pre‐adapted medium; pre‐adapted medium = "conditioned" medium that contains growth factors and reduces costs), and discard the remaining supernatant.
4 Resuspend and pool the pelleted cells into fresh complete J‐MEM so that after addition of virus P_2_ stock (next step) the concentration will be ≤2 × 10^7^ cells/ml.
  Try to keep the cell concentration as close as possible to 2 × 10^7^ cells/ml, without going over.
5 Add virus at an MOI of ∼5 PFU/cell.
6 Adsorb virus for 1 hr at room temperature with periodic swirling.
7 During adsorption, complete the glass roller bottle medium:
  - Add 700 ml fresh complete J‐MEM, 11 ml 100× penicillin‐streptomycin solution, and 1.5 ml 1000× amphotericin B working solution.
  - Place into a 33°C spinning water bath to equilibrate.
8 After the 1 hr adsorption, pour infected cell suspension into pre‐equilibrated roller bottle.
  The final cell concentration should not exceed 6.5 × 10^5^ cells/ml.
9 Incubate, with spinning, ∼65 hr at 33°C.
  Alternatively, if checking incubation time by hemocytometer counting, incubate until ∼40% of the cells remain alive.
10 After incubation, pellet cells using a refrigerated centrifuge in 250‐ml centrifuge tubes 30 min at ∼500 × *g*.
  Pour supernatant into collection container and add bleach to supernatant before discarding.
11 Resuspend pelleted cells in 22 ml of HO buffer. Note the final volume of resuspended cells (should be 24 to 26 ml) and transfer into two 30‐ml COREX tubes.
  The suspension may be frozen and stored at –80°C at this step, or used immediately for purification.

#### Perform virus purification

12 Thaw HO suspension if frozen, and place tubes on ice.
  The suspension MUST be kept cold through the following steps.
13 Sonicate suspension sample to disrupt cells.
  Use a small probe at a mid‐range setting for 10 s.
14 Add 1/50th of the total volume recorded in step 11 of 10% DOC to each tube. Cover tubes with Parafilm and vortex the suspension 3 to 5 s.
15 Let sit for 5 min, then vortex 3 to 5 s. Let the tubes sit for an additional 25 min.
16 Add 2/5th of the total volume of Vertrel‐XF to each tube and sonicate to mix the two layers and create an emulsion.
  Use a small probe at a mid‐range setting for 30 s.
17 Add the same amount of Vertrel‐XF as in step 16 to each tube and sonicate again at the same setting.
  The total volume of Vertrel‐XF must be less than the aqueous volume to make an emulsion.
18 Centrifuge suspension 10 min at ∼9000 × *g* in a refrigerated Super‐speed centrifuge.
19 Remove the top aqueous layers into fresh 30‐ml COREX tubes with a plastic pipet, making sure to note the new volume.
20 Add 9/10th the total volume noted above of Vertrel‐XF to each tube and re‐emulsify.
21 Centrifuge again 10 min at ∼9000 × *g* to separate phases.
22 During final phase separation, prepare a 1.2 g/ml and 1.44 g/ml cesium chloride gradient(s) in an SW‐28 ultracentrifuge tube.
  Cesium chloride solutions are filter sterilized and may be stored at room temperature for years. Gradients are prepared fresh for each use.
  A SW‐28 ultracentrifuge tube holds ∼38 ml of fluid. Since the combined aqueous phases after Vertrel‐XF purification represent ∼22 ml, the cesium chloride gradient is made by pipeting 8 ml of the 1.44 g/ml solution into the bottom of the tube and then pipeting 8 ml of the 1.22 g/ml solution on top. Effective gradient formation is achieved by initially pipeting the 1.2 g/ml solution "roughly" into the 1.44 g/ml solution to cause mixing. This is followed, as more and more 1.2 g/ml solution is added, by adding the 1.2 g/ml solution progressively more and more carefully so there is little mixing by the time the final few milliliters of 1.2 g/ml solution are added on top.
23 Remove the aqueous phases from step 21 with a fresh pipet and layer contents of both COREX tubes onto a single 1.2‐1.44 g/ml cesium chloride gradient.
24 Centrifuge gradient ≥5 hr at ≥87,000 × *g* in an ultracentrifuge at ∼5°C.
25 Collect the lower virus band by top, bottom, or side puncture into a sterile tube.
  You may also collect the upper "top component," which contains empty, genome‐deficient, virus particles.
26 Pipet collected virus band into prepared dialysis tubing and dialyze against three sets of dialysis buffer (D‐buffer or 2× SSC) for ≥1 hr, then ≥3 hr, then ≥6 hr at ∼5°C.
  CAUTION: Sodium azide is toxic.
  Dialysis tubing may be purchased prepared in sodium azide from several suppliers. Alternatively, dry dialysis tubing may be prepared as described in Sambrook et al. (1989).
27 Collect the virus into an appropriate vessel and store at 4°C, or add glycerol to 15% and freeze at –80°C.
  If freezing, make sure to check OD_260_ before the addition of the glycerol.
  1 OD**_2_**_60_ = 2.1 × 10^12^ reovirus particles = 0.370 mg virus protein/ml (Coombs, 1998a; Smith et al., 1969).
  OD_260_/OD_280_ = 1.4‐1.6 for intact virus.
  OD_260_/OD_280_ = 1.1‐1.3 for top component.

## Basic Protocol 2 QUANTIFICATION OF MAMMALIAN REOVIRUSES BY PLAQUE ASSAY WITH NEUTRAL RED STAINING

Mammalian reoviruses cause cytopathic effect (CPE) and eventual death of host cells. Because of this, infected monolayers will show zones of lysed cells (called plaques), which are essentially areas of infected cells. A plaque assay is used to quantify the amount of infectious virus in a sample, indicated by the number of plaques formed on a monolayer of mouse L929 cells. If the dilution of the virus sample is high enough, the plaque represents a single infectious particle that has infected a cell and continued to spread to the adjacent cells. Using this method of quantification, a virus titer can be calculated to units per milliliter. This particular plaque assay uses a neutral red stain to enhance visibility for counting purposes. The neutral red assay technique is also used to replenish P_0_ virus stocks when these stocks run low (see Basic Protocol 1). Repeated passages should be performed at low multiplicities of infection to avoid generation of defective interfering particles.

### Materials

Pre‐attached L929 cell monolayers in 12‐ or 6‐well plates or 60‐mm dishes (see Support Protocol 2)
Gel saline (see recipe)
Mammalian reovirus stock/sample to be titrated (the virus may be initially obtained from the ATCC or clinical samples)
2% (w/v) agar (Difco Bacto)
Complete 2× Medium 199 (M‐199) (see recipe)
Amphotericin B working solution, 1000× (see recipe)
2% (w/v) neutral red solution (in dH_2_O)
2× PBS (Current Protocols, 2006)
Penicillin‐streptomycin solution, 100× (see recipe), optional

Dilution tubes
Micropipettors
100‐ to 1000‐µl pipet tips
20‐ to 200‐µl pipet tips
10‐ml pipets
Vortex
Autoclavable waste container
Microwave
62°C water bath

#### Day 0

1 Set up L929 cells in appropriate well plates or dishes and incubate overnight at 37°C (see Support Protocol 2).
  Set up 12‐well plates if larger ranges of dilutions need to be tested, for example in samples where the titer is completely unknown. 6‐well plates can be set up if the general titer is known.

#### Day 1

**Figure 2:**
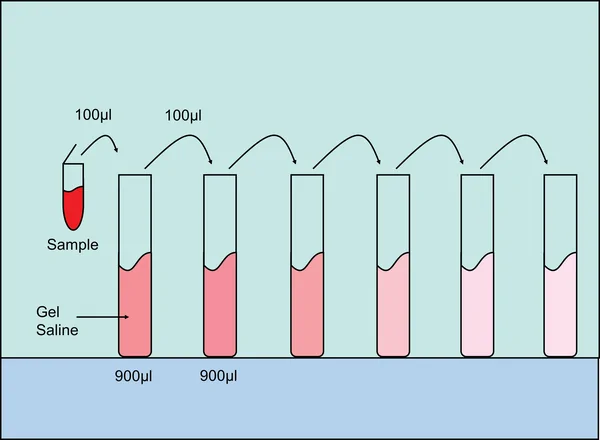
Diluting unknown virus sample for plaque assay.

2 Prepare a set of six dilution tubes for each sample by pipetting 900 µl of gel saline into each tube.
  This can be done the previous day if many samples are being titered, and stored at 4°C overnight.
3 Make six serial 1:10 dilutions of the samples to be titered in gel saline as follows:
  - Vortex the sample, then pipet 100 µl of the sample into the first dilution tube, giving a 1 × 10^–1^ dilution.
  - Discard the used pipet tip into an autoclavable waste container.
  - Vortex the 1 × 10^–1^ dilution tube, then with a fresh pipet tip, pipet 100 µl of the 1 × 10^–1^ dilution into the next dilution tube, giving a 1 × 10^–2^ dilution.
  - Perform this for all dilution tubes for each sample (Fig. 2), using a fresh pipet tip for each dilution.
    For high‐titered samples, start with a single 1:100 dilution, giving a 1 × 10^–2^ dilution, followed by five 1:10 dilutions. In this case, the first dilution tube contains 10 µl of sample and 990 µl of gel saline.

4 Dump out medium from the 6‐well (and/or 12‐well) plates that contain preformed L929 cell monolayers and inoculate 100 µl of the three highest dilutions onto cells, in duplicate, making sure to vortex each dilution tube first. Label wells with dilution factor used.
  A single pipet tip may be used if the highest dilution is inoculated first, followed by the next, etc.
  For 12‐well plates, all six dilutions can be used in duplicate. Alternatively, all six dilutions may be used a single time in a 6‐well plate, but for most accurate results, duplicate platings are preferred.
5 Allow virus to adsorb on cell monolayer by incubating 1 hr at room temperature.
  Gently rock flask every 10 to 12 min to disperse liquid overlay and prevent any regions of the monolayer from drying out.
6 While waiting for virus to adsorb, melt the 2% agar in a microwave and place in a 62°C water bath.
  Calculate sufficient volume to allow overlay of all wells to be used.
  Agar is best melted by using 50% power, and swirling intermittently to ensure no solid clumps are present. The cap of the agar should be twisted slightly open prior to heating, to prevent the potentially explosive build up of hot gasses.
7 Allow the agar to cool to 62°C in the water bath.
8 Just before 1 hr adsorption time is up, prepare plaque assay overlay by adding an equal volume of room temperature 2× complete M‐199 (with 6% FBS) into the bottle containing 62°C agar. Swirl to mix.
  To ensure stock medium remains sterile, prepare plaque assay overlay in a sterile biosafety cabinet. To keep agar mixture warm, the bottle can be kept in a container half‐filled with hot tap water.
9 Add 1/1000^th^ the total volume of 1000× amphotericin B working solution to the agar/199 mixture.
10 Overlay each well with 3 ml of complete agar/199 mixture for 6‐well plates, or 1.25 ml for each well in a 12‐well plate. Allow agar to solidify at room temperature for ∼10 min.
  Set aside as stacks of no more than two plates high, and do not move plates until agar has solidified.
11 Put plates in a 37°C incubator. Incubate plates for 3 days.

#### Day 4

12 Prepare fresh agar/M‐199 overlay mixture, as described above.
13 “Feed” assay by adding 2 ml of overlay into each well of a 6‐well plate, or 0.75 ml of overlay into each well of a 12‐well plate.
14 Return plates to a 37°C incubator. Incubate plates for 3 days.

#### Day 7

15 Prepare neutral red staining solution by melting the 2% agar in a microwave and placing in a 62°C water bath, as described above.
16 Allow agar to cool to 62°C.
17 Add 2 ml of 2% neutral red solution per 50 ml agar into warm agar.
  To ensure the stock medium remains sterile, prepare plaque assay overlay in sterile biosafety cabinet. To keep agar mixture warm, the bottle can be kept in a container half‐filled with hot tap water.
18 Add an equal volume (to agar) of 2× PBS into agar/neutral red solution.
19 If planning to pick plaques for P_0_ virus stock (see Basic Protocol 1), add 1/100^th^ the total volume of 100× penicillin/streptomycin solution and 1/1000^th^ the total volume of 1000× amphotericin B working solution to the agar/PBS/neutral red solution.
20 Stain assay by adding 2 ml of staining solution into each well of a 6‐well plate, or 0.75 ml into each well of a 12‐well plate.
21 Return plates to a 37°C incubator.

#### Day 8

**Figure 3:**
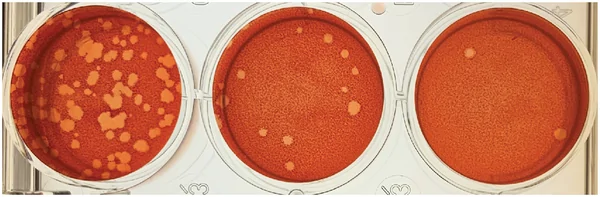
Neutral red‐stained reovirus plaques. Plaques are clear circular areas within red‐stained cell monolayers. Ten‐fold (1:10) serial dilutions of the same stock virus are shown from left to right.

22 Count plaques within 18 to 24 hr of adding the staining solution.
  Plaques should be seen as a circular lighter color area within the well (Fig. 3). The arrows indicate individual plaques.

23 Count each plaque as one “plaque‐forming unit” or PFU.
  For best results, count wells that contain 20 to 200 plaques. Plaques may be easier to see by placing the plate onto a light box.
24 Calculate virus titer by taking into account the dilution factor.
  Plaques are counted and averaged between the duplicate wells, giving a number (N) between 20 and 200. If this number is found in the wells inoculated with the dilution tube 1 × 10^–5^, then the titer is N × 10^6^; that is, the reciprocal of the dilution factor (N × 10^5^), multiplied by 10 (because 1/10 ml inoculated).

## Alternate Protocol 2 QUANTIFICATION OF MAMMALIAN REOVIRUSES BY PLAQUE ASSAY WITH CRYSTAL VIOLET STAINING

**Figure 4:**
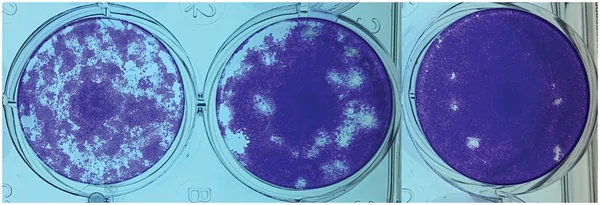
Crystal violet‐stained reovirus plaques. Plaques are clear circular areas within blue‐stained cell monolayers. Ten‐fold (1:10) serial dilutions of the same stock virus are shown from left to right.

Plaques should be counted within 24 hr after staining with the neutral red staining technique (see Basic Protocol 2). However, a crystal violet staining method can be used instead for a stable assay that can be kept indefinitely (Fig. 4). This method kills both cells and virus, and it can therefore not be used when plaques need to be picked for replenishing P_0_ virus stocks.

### Additional Materials (also see Basic Protocol 2)

2% (v/v) formaldehyde solution (37% formaldehyde in ddH_2_O)
Distilled water
2% (w/v) crystal violet stain (in methanol)

Small metal scoop
Paper towels

#### Days 0 to 4

1 Perform steps 1 to 14 of Basic Protocol 2.

#### Day 7

2 Fix cells in each well by pipeting 2% formaldehyde solution to cover agar. Leave on for > 1 hr (or overnight).
3 Gently scoop out agar plug from each well with a metal scoop.
  Make sure not to scrape off cell monolayer, or twist agar inside well, which may cause regions of the monolayer to lift off.
4 Re‐fix cells by pipeting 2% formaldehyde solution to cover cell monolayer. Leave on for ∼10 min.
5 Dump out formaldehyde solution, shaking once or twice to expel residual solution from the wells.
6 Gently wash wells once with distilled water.
  Squirt dH_2_O onto the side of each well to avoid pouring water directly on the cell monolayer, which may cause the monolayer to lift off.
7 Shake out remaining water from wells and add enough crystal violet solution to cover the bottom of each well.
8 Let crystal violet solution sit for at least 30 min.
  The longer the incubation period with the crystal violet stain, the greater the staining intensity achieved.
9 Pipet out the crystal violet stain, putting it back into the stock solution for re‐use.
10 Rinse off excess crystal violet stain from the wells under tap water, at an angle.
11 Place plates inverted onto paper towels, and leave to dry before counting plaques.
  Apply the same counting technique as in Basic Protocol 2. Crystal violet–stained plates will keep indefinitely.

## Basic Protocol 3 STORAGE OF MAMMALIAN REOVIRUSES

**Figure 5:**
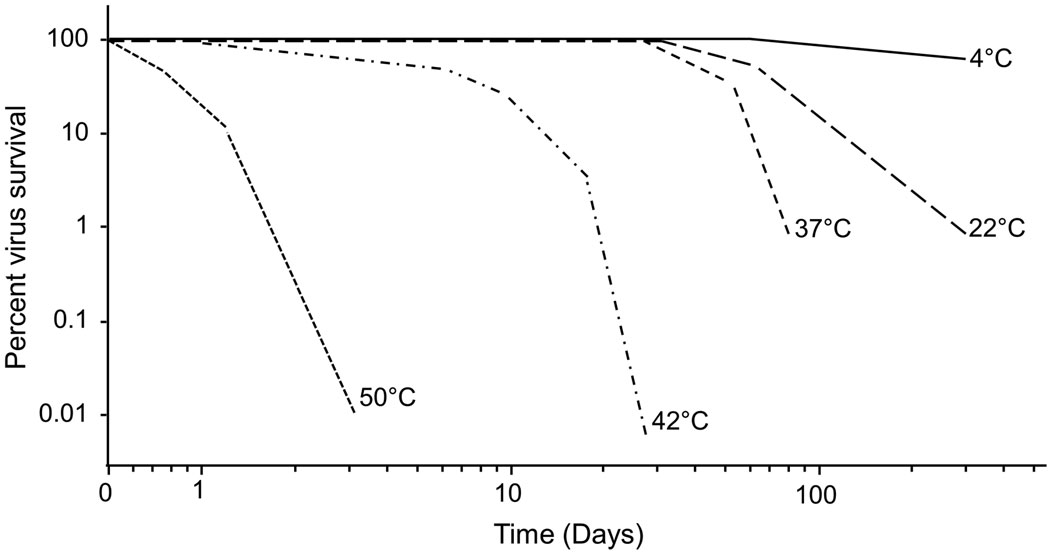
Infectious reovirus survival when stored at various temperatures.

Mammalian reoviruses are fairly stable at 4°C, and they can be kept there for many months with little, if any, decline in infectivity (see Fig. 5). The virus also maintains high infectivity at room temperature (∼22°C) for about a month, but it loses infectivity more rapidly at higher temperatures (Fig. 5). Large stocks of virus may also be divided into aliquots and kept frozen (less than –70°C) for years.

## Support Protocol 1 GROWTH AND MAINTENANCE OF MOUSE L929 CELLS

Although mammalian reoviruses may infect many different types of cell lines, mouse L929 cells are the preferred cell line used to efficiently amplify and quantify the virus. This protocol describes how to maintain the L929 cell line for use in Basic Protocols 1, 2, and 3 in either a monolayer or suspension form.

### Materials

Mouse L929 cells grown in either 25‐, 75‐, or 150‐cm^2^ tissue culture flask, or in a suspension culture flask (see Basic Protocol 1)
PBS/EDTA (see recipe)
Trypsin (see recipe)
Complete J‐MEM (see recipe)

5‐ml and 10‐ml pipets
500‐ to 1000‐ml glass flat‐bottom “Florence” flask (for suspension culture)
Additional reagents and equipment for counting cells using a hemacytometer (Stevenson, 2006)

*NOTE*: All solutions and materials coming into contact with cells must be sterile, with proper aseptic techniques used.

*NOTE*: All cell culture incubations utilize a 37°C incubator at 5% CO_2_, except the suspension cultures, which are maintained in a 37°C non‐CO_2_ incubator.

#### Prepare monolayer cultures

**Figure 6:**
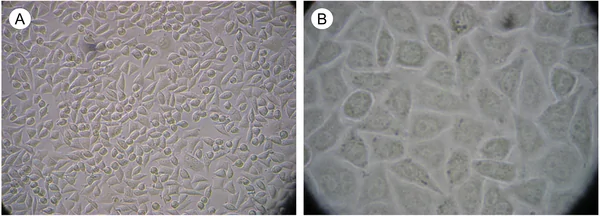
L929 cells at (**A**) 100× and (**B**) 400× magnification.

The steps that follow are based on treating a single 150‐cm^2^ monolayer tissue culture flask so that it regains confluency in 2 days. We routinely split L929 cells 1:5 on Mondays and Wednesdays, and 1:10 on Fridays. The single 150‐cm^2^ flask may be scaled up or down as anticipated needs dictate. In general, cells can be split ∼1:2.5 to be nearly confluent (∼90% to 95% confluent; optimal for virus growth; see Fig. 6) the next day; 1:5 to be confluent in ∼2 days; or 1:10 to be confluent in ∼3 days.

1a Pour (or pipet) out old medium from confluent tissue culture flask, saving ∼10 ml in a sterile tube as “pre‐adapted” medium.
2a Rinse monolayer with ∼10 ml of PBS/EDTA. Pipet out PBS/EDTA.
3a Add 2 ml of fresh PBS/EDTA/trypsin (at a final trypsin concentration of 2.5 mg/ml) into flask. Rock flask back and forth 2 to 3 times to completely cover bottom with trypsin mixture. Remove trypsin mixture.
4a Put flask into a 37°C incubator for ∼10 min. After the time has elapsed, check for loosening of cells under a microscope.
  Gentle tapping of the bottom of the flask may help detach cells.
5a Pipet 5 ml of pre‐adapted J‐MEM (from step 1a) into trypsinized flask and titurate to wash cells off the bottom of the flask and break up clumps. Tituration is performed by placing the pipet against the flat bottom of the flask and expelling liquid to break up large cell clumps.
6a Remove all but 1 ml of cell suspension.
  The remaining 4 ml may be discarded or used to set up additional flasks or plates for further applications (see Support Protocol 2).
7a Add the remaining 5 ml of pre‐adapted completed J‐MEM (from step 1a) plus 24 ml of fresh medium.
8a Place the flask back into the incubator.
  Plastic tissue culture flasks may be re‐used up to about a dozen times. If re‐using plastic flasks, replace with new flasks every ∼12 splittings.
  To ensure cells remain susceptible to infection, discard cells after high passages (∼6 months). Establish a new line from a low‐passage frozen stock, which should be kept at –135°C or in liquid N_2_.

#### Prepare suspension cultures

The steps that follow are based on 500 ml of cells kept in a 1000‐ml flat‐bottom glass “Florence” flask. These cells are checked each day and split back to a concentration of ∼5 × 10^5^ cells/ml as follows:

1b With a sterile pipet, remove ∼1 ml of cell suspension from the flask containing mouse L929 cells.
2b Count the cell concentration using this 1 ml cell suspension in a hemacytometer (Stevenson, 2006) *.*
3b Calculate required amount of cell suspension needed to reduce stock solution to 5 × 10^5^ cells/ml.
4b Pour out extra cells into a sterile container to either discard or plate for use in further applications (see Support Protocol 2).
5b Add fresh complete J‐MEM into the flask to return the volume to 500 ml and the cells to a concentration of ∼5 × 10^5^ cells/ml. Place the flask back into the incubator.
  Cells may occasionally be split to 2.2 × 10^5^ cells/ml and left for 2 days (i.e., over weekends and holidays).
  Replace the glass Florence flask with another chromic acid–cleaned sterile one weekly.

## Support Protocol 2 PLATING L929 CELLS

This protocol describes plating L929 cells for quantification, purification, or amplification.

### Materials

L929 cells in suspension (see Support Protocol 1)
Complete J‐MEM (see recipe)

5‐ and 10‐ml pipets
Appropriate‐sized vessels

1 For use the next day, dilute cells to 4.2–4.5 × 10^5^ cells/ml with complete J‐MEM.
2 Pipet cells depending on their use:

**Table:** 

To titer virus stocks/samples:	2.5 ml/well in 6‐well cluster dishes
	1.25 ml/well in 12‐well cluster dishes
To pick plaques:	5 ml/60‐mm tissue culture dish
To set up virus growths:	25‐cm^2^ flask—5 ml
	75‐cm^2^ flask—15 ml
  Other‐sized vessels (i.e., 24‐well or 96‐well plates) may also be used; scale appropriately.
3 Incubate plates/flasks overnight for use the next day.
  Alternatively, cells may be set up at greater densities, at 7–9 × 10^5^ cells/ml, for use later the same day; or set up at lower densities, at 2 × 10^5^ cells/ml, for use 2 days later. However, cells to be set up for use the same day should be set up only from suspension cultures. Trypsinized cells normally need to regenerate for ≥24 hr to allow efficient virus entry and replication.

## REAGENTS AND SOLUTIONS

*Use deionized, distilled water in all recipes and protocol steps*.

### Amphotericin B (1 mg/ml), 1000× (working solution)

1 ml of 20,000× amphotericin B solution (see recipe)
Using aseptic techniques, bring to 20 ml with sterile ddH_2_O
Divide into 10‐ml aliquots into sterile tubes and store for several years at –20°C

### Amphotericin B (20 mg/ml), 20,000× (stock solution)

100 mg amphotericin B solubilized powder, γ‐irradiated (Sigma, cat. No. A9528)
Using aseptic technique, reconstitute amphotericin B power in 5 ml sterile ddH_2_O
Divide into 1‐ml aliquots into sterile cryovials and store for several years at –20°C

### Cesium chloride gradient solutions

*1.2 g/ml*:

33.3 g CsCl
1 ml 1 M Tris·Cl, pH 7.4 (Current Protocols, 2006), dissolved in 100 ml ddH2O
Adjust refractive index to 1.3533 with 10 mM Tris·Cl, pH 7.4

*1.44 g/ml*:

67 g CsCl
1 ml 1M Tris·Cl, pH 7.4 (Current Protocols, 2006), dissolved in 100 ml ddH2O
Adjust refractive index to 1.378 with 10mM Tris·Cl, pH 7.4
Filter sterilize with a 0.2‐µm membrane
Store for several years at room temperature

### Dialysis buffer

30 ml 5 M NaCl (150 mM)
15 ml 1 M MgCl_2_ (15 mM)
10 ml 1 M Tris·Cl, pH 7.4 (Current Protocols, 2006)
Bring to 1 L with ddH_2_O
Store up to 6 months at room temperature

### Gel saline

8 g NaCl
0.03 g CaCl_2_
0.17 g MgCl_2_·6H_2_O
1.2 g H_3_BO_3_
0.05 g Na_2_B_4_O_7_·10H_2_O
3 g gelatin, type A
Heat and stir to dissolve in 1 L ddH_2_O
Autoclave
Store for several years at 4°C

### l‐Glutamine (100×)

14.6 g l‐glutamine
Bring to 500 ml with ddH_2_O
Filter sterilize with a 0.2‐µm membrane
Aliquot into sterile 100‐ml bottles, and cool to 4°C overnight
Place bottles at –20°C and swirl bottles every 30 min until frozen
Store for several years at –20°C

### HO buffer

1 ml 1 M Tris·Cl, pH 7.4 (10 mM final concentration) (Current Protocols, 2006)
5 ml 5 M NaCl (250 mM final concentration)
67 µl 2‐mercaptoethanol (10 mM final concentration)
Bring solution to 100 ml with ddH_2_O and filter sterilize with a 0.2‐µm membrane
Store for several years at 4°C

### J‐MEM (for culturing L929 cells), complete

940 ml 1 × S‐MEM (Joklik's S‐MEM; e.g., SAFC Biosciences, cat. No. 56449C)
50 ml tissue‐culture grade fetal bovine serum (FBS)
10 ml l‐glutamine stock solution (see recipe)
Store up to 1 year at 4°C
  Alternatively, if using a 2× S‐MEM solution, use only 500 ml and add sterile ddH_2_O to final volume of 1 L.

### Medium 199 (M‐199) (6% FBS); 2×, complete

900 ml 2× 199 medium (made at double concentration; Medium 199 Modified; SAFC Biosciences, cat. No. 51312C or Invitrogen, cat no. 31100‐019)
60 ml fetal bovine serum (FBS)
20 ml l‐glutamine stock solution (see recipe)
20 ml penicillin‐streptomycin stock solution (see recipe)
Store up to 1 year at 4°C

### PBS/EDTA

8 g NaCl
0.2 g KCl
1.15 g Na_2_HPO_4_
0.2 g KH_2_PO_4_
0.2 g EDTA
Dissolve in 1 L of ddH_2_O
Autoclave
Store for several years at room temperature

### Penicillin‐streptomycin solution, 100×

1.2 g penicillin G
2.0 g streptomycin sulfate
Bring solution to 200 ml with ddH_2_O and filter sterilize with a 0.2‐µm membrane
Aliquot in 100‐ml bottles and store for several years at –20°C

### Trypsin (25 mg/ml), 10×

1.25 g trypsin (1:250; porcine pancreas, Sigma)
0.425 g NaCl
Bring to 50 ml with 1× PBS (Current Protocols, 2006) and filter sterilize with a 0.2‐µm membrane
Aliquot into desired amounts and store for several years at –20°C

## COMMENTARY

### Background Information

The mammalian reoviruses (MRV) are the prototypical members of the virus family *Reoviridae*. Members of this family have a genome of 9 to 12 segments of double‐stranded (ds) RNA that are surrounded by 2 to 3 concentric, non‐enveloped protein capsids (Dermody et al., 2013). The *Reoviridae* family contains the only group of dsRNA viruses (out of seven current dsRNA virus families) that infect mammals. The *Reoviridae* family currently consists of 15 recognized genera [NCBI Taxonomy Browser: Taxonomy browser (Reovirales), nih.gov], and a variety of other newly discovered viruses that share key features (dsRNA segmented genome, non‐enveloped, and capacity to infect mammals) supports inclusion of additional currently unclassified agents (*i.e., Cimodo virus*). In addition to the genus *Orthoreovirus, the Reoviridae* includes *Rotavirus*, agents responsible for a significant amount of viral gastroenteritis and numerous deaths annually worldwide, the economically important insect‐vectored *Orbivirus*, and a variety of other viruses that infect animals, fungi, and plants.

The *Orthoreovirus* genus is divided into three subgroups: non‐syncytia‐inducing mammalian reovirus (subgroup 1), avian reovirus and Nelson Bay virus (subgroup 2), and baboon reovirus (subgroup 3). The Orthoreoviruses have a genome of 10 segments of dsRNA surrounded by 2 concentric protein capsids. MRV are represented by three reovirus serotypes: type 1 Lang (T1L), type 2 Jones (T2J), and type 3 Dearing (T3D). A variety of other strains are placed into one of these three serotypes, based primarily upon hemagglutination characteristics. The complete genomic sequences of all 10 genes of all 3 prototype MRV have been determined. The orthoreovirus genome consists of 3 large segments (L1, L2, L3) ranging in size from ∼3.8 to 4.0 kilobase pairs (kbp), 3 medium segments (M1, M2, M3) ranging in size from ∼2.0 to 2.3 kbp, and 4 small segments (S1, S2, S3, S4) ranging in size from ∼1.0 to 1.6 kbp (total genome size ∼23.5 kbp) (Breun et al., 2001; Wiener et al., 1989; Yin et al., 2004).

The L genome segments encode large proteins [usually named lambda (λ)], which are found in the inner virus capsid (called *core*) and which have a variety of enzymatic roles associated with the generation of mRNA from the enclosed genome (Dryden et al., 2008; Sandekian & Lemay, 2015). Proteins λ1 and λ2 have major structural roles; λ1 constitutes the core shell and protein λ2 forms pentameric “turrets” at each of the core's icosahedral vertices. Protein λ3 serves as the viral RNA‐dependent RNA polymerase (RdRp) and is located under each turret (Zhang et al., 2004). The core remains intact throughout the viral replicative cycle, which takes place in the infected cell's cytoplasm. Thus, the RdRp, which is located inside the virus capsid along with the genomic dsRNA, must have sufficient freedom of movement to read the template dsRNA to transcribe mRNA. This also implies that the core capsid must have holes in it to allow transport of nucleotides and other components into the core interior. In addition to the three λ proteins, orthoreovirus cores are composed of two additional proteins. These are a medium protein [named mu (µ); specifically µ2] and a small protein [named sigma (σ); specifically σ2]. The σ2 protein appears to serve as a “clamp” to help hold the core capsid together. The µ2 core protein appears to serve as an RdRp cofactor since expressed RdRp has little, if any, polymerase activity by itself (Starnes & Joklik, 1993). The core is surrounded by an outer capsid made up of three additional proteins. The major outer capsid protein is µ1 which, as a trimeric aggregate, forms the icosahedral T = 13 lattice. The µ1 outer capsid lattice is decorated with three copies of another major outer capsid protein (σ3) associated with each µ1 trimer. The outer capsid also contains a smaller number of another σ protein (σ1), which serves as the cell attachment protein. The related avian orthoreoviruses possess the same proteins, although they are given different names.

The development and refinement of a “Reverse genetics” system (Kobayashi et al., 2007; 2010) for reovirus has allowed further refinement of molecular signals for numerous functions performed by various reovirus proteins. These include tagging viral proteins for the visualization of “viral factories” (Bussiere et al., 2017), determining amino acid residues responsible for plaque size and oncolytic potential (Mohamed et al., 2020), and determining amino acid residues responsible for the temperature‐sensitive phenotype of certain mutants (Glover et al., 2021).

Reoviruses infect a wide range of cells, both in vitro and in vivo. The virus usually infects specialized intestinal epithelial cells (M cells) that overlie Peyer's patches in vivo. The virus then migrates between and/or through the M cells into mucosal mononuclear cells in the Peyer's patch, and subsequently into a large number of extraintestinal sites, including heart, liver, and the central nervous system. The onset of cell infection is initiated by the interaction of the σ1 cell‐attachment protein with an appropriate cell surface protein. Evidence points to junction adhesion molecules and/or sialyated proteins as possible receptors. Once bound, the virus is internalized. For reoviruses, this seems to involve receptor‐mediated, low‐pH‐driven proteolysis, which serves to remove the σ3 outer capsid protein, and also results in clipping of the µ1 outer capsid protein into two peptides [called delta (δ) and phi (φ)]. The resulting particle [called Intermediate (or Infectious) Subviral Particle (ISVP)] can also be produced in the laboratory and is capable of directly penetrating membranes. Once exposed to the cellular milieu, the ISVP loses the rest of its outer capsid proteins and peptides to generate the core particle. Nascent mRNA produced from cores serves both for protein translation and also as a template to regenerate progeny dsRNA genomes. The progeny dsRNA genomes are assembled by an efficient process into sets of the 10 different genes, which then become progressively coated by newly translated viral proteins to produce progeny virus. Progeny virus is released when the infected cells lyse, a process that generally occurs 24 to 72 hr post‐infection.

MRV infect a wide range of animals, including humans. Serologic evidence indicates >90% of tested human populations have antibodies to MRV by the time they reach adulthood. In humans, MRV infection is usually asymptomatic. There are sporadic reports of cold‐like symptoms and more serious illnesses, including hepatic, and possibly neuronal, involvement (Hermann et al., 2004; Johansson et al., 1996; Tyler et al., 2004). The segmented nature of the viral genome allows genetic mixing when two different strains of the same virus infect the same cell. This genetic mixing (analogous to influenza virus *genetic shift*) leads, as in the case of influenza virus, to the generation of antigenically distinct virus clones, which may not initially be recognized by the human immune system.

### Critical Parameters and Troubleshooting

Mammalian reoviruses generally are easily grown and achieve high titers. They also are among the most stable of known viruses, which, when combined with their general safety, makes their manipulation generally straightforward. As indicated earlier, they are stable when stored at refrigerator temperatures. The avian reoviruses are less stable than the mammalian viruses. Reoviruses also are stable under acidic conditions, and they will survive passage through the gastrointestinal system, their normal route of infection. However, the viruses are sensitive to alkaline conditions and will lose infectivity rapidly at pH values >9. Thus, solutions and media require buffering.

Virus growth is dependent upon healthy cells. Although the precise cell cycle stage of cells is not critical, the virus grows best in actively growing cells. Thus, optimum results are obtained with cells that are 90% to 98% confluent. Viruses will grow (and plaque) in cells that have just become confluent, but yields (and plaquing capacity) are reduced if cell monolayers have been confluent for >12 hr. Similarly, since optimal cell health is necessary, great care should be taken during plaque assays that the agar overlay is not too hot. We have found that mixing equivalent volumes of 62°C 2% agar with room‐temperature (∼22°C) 2× medium, by adding the medium into the bottle that contains the agar, results in a solution that is 43° to 45°C. Be sure to “wrist test” the mixture's temperature, and, if necessary, allow it to cool sufficiently so as to not “cook” the cells. Plaque assays performed in 12‐well plates are especially sensitive to the volume of agar/medium added. The amounts specified in the above protocols should not be exceeded. If they are, plaque development and staining suffer markedly.

Like other RNA viruses, reoviruses have the capacity to mutate more rapidly than many DNA viruses. In addition, defective interfering particles may arise after repeated high multiplicity infections. Thus, it generally is a good idea not to exceed four passages of virus amplification. Low passage stocks may be replenished by picking fresh plaques. Common problems, and their solutions, are indicated in Table 1.

**Table 1:** Troubleshooting Guide for Plaque Assays and Reovirus Purification

Problem	Possible cause	Solution
No visible plaques	Dilution series not appropriate	Use a wider range of dilutions, from 10^0^ – 10^‐9^
No visible plaques	Agar overlay too hot	“Wrist test” to confirm solution not too hot
Plaques too large or too small	Incubation time too long or too short	Adjust incubation tme
Plaques appear as “comets” instead of being round	Plates moved before agar set	Overlay plates with agar and set down immediately without disturbing for at least 5 min
VertrelXF does not emulsify	Too much VertrelXF	Ensure the volume of VertrelXF is less than the volume of solution being extracted; keep sonicated sample chilled

### Understanding Results

#### Plaque assays

Plaques will present as clear or “milky” circular areas in the cell monolayers. They may be visible unstained, but enumeration is easier if the cell monolayer has been stained. Plaques normally contain ∼10^5^ infectious viruses; thus, there is sufficient virus in a picked plaque (passage zero; P_0_) to amplify as described herein.

#### Virus passaging

The titer of virus usually increases 10× to 100× with each of the first two passages. Final titers obtained are dependent upon the virus strain. In our hands, MRV T1L regularly grows to titers of ∼10^9^ PFU/ml, T2J to titers of ∼4 × 10^7^ PFU/ml, and T3D to titers of 3‐8 × 10^8^ PFU/ml in L929 cells.

#### Virus purification

Mammalian reovirus grows well in L929 cells grown either as monolayers or as suspension cultures. For large‐scale growth, it is easier to work with suspension cultures of 1 L or more. The amount of virus obtained is also strain‐dependent; several milligrams of purified virus can usually be obtained from 1 L of infected cells. The centrifugation medium (cesium chloride) should be removed by dialysis before the virus is used.

#### Virus storage

Reoviruses are among the most stable of viruses. Nevertheless, their titer will decrease with time. The rate of infectivity decrease is temperature‐dependent (see Fig. 5). Virus stocks will lose detectable infectivity if stored at 4°C for many years. Similarly, frozen virus stocks will also lose some infectivity after prolonged storage. Thus, periodic stock replenishment is needed.

### Time Considerations

#### Plaque assays

The standard MRV plaque assay in 6‐well plates takes ∼1 week when performed at 37°C. Temperature‐sensitive clones (see Coombs, 1998b) take ∼50% longer if performed at a lower “permissive” temperature of 31°C to 33°C. When these assays are performed in 12‐well plates, plaques develop slightly faster, and they may be visualized ∼1 day earlier.

#### Virus passaging

Completion of the P1 passage normally takes 5 to 10 days for sufficient CPE (>85%) to develop. Because the titer increases with each passage, P_2_ usually develop sufficient CPE within 3 to 5 days. The process of freeze‐thawing flasks 3 times to aid release of virus from infected cells normally takes a minimum of an additional day.

#### Virus purification

Growth of virus takes ∼65 hr. Processing to obtain a cesium chloride–purified virus band will take most of an additional day, and dialyzing the virus to remove the cesium chloride will take 1 to 2 additional days.

### Author Contributions

**Kevin M. Coombs**: Conceptualization, formal analysis, funding acquisition, investigation, methodology, project administration, supervision, visualization, writing – original draft, writing – review & editing.

### Conflict of Interest

The author declares no conflict of interest.

## Data Availability

All data are contained herein
